# A Fast and Scalable Workflow for SNPs Detection in Genome Sequences Using Hadoop Map-Reduce

**DOI:** 10.3390/genes11020166

**Published:** 2020-02-05

**Authors:** Muhammad Tahir, Muhammad Sardaraz

**Affiliations:** Department of Computer Science, COMSATS University Islamabad, Attock Campus 43600, Pakistan; m_tahir@cuiatk.edu.pk

**Keywords:** DNA, NGS, SNP, Hadoop, Map-Reduce, accuracy, execution time

## Abstract

Next generation sequencing (NGS) technologies produce a huge amount of biological data, which poses various issues such as requirements of high processing time and large memory. This research focuses on the detection of single nucleotide polymorphism (SNP) in genome sequences. Currently, SNPs detection algorithms face several issues, e.g., computational overhead cost, accuracy, and memory requirements. In this research, we propose a fast and scalable workflow that integrates Bowtie aligner with Hadoop based Heap SNP caller to improve the SNPs detection in genome sequences. The proposed workflow is validated through benchmark datasets obtained from publicly available web-portals, e.g., NCBI and DDBJ DRA. Extensive experiments have been performed and the results obtained are compared with Bowtie and BWA aligner in the alignment phase, while compared with GATK, FaSD, SparkGA, Halvade, and Heap in SNP calling phase. Experimental results analysis shows that the proposed workflow outperforms existing frameworks e.g., GATK, FaSD, Heap integrated with BWA and Bowtie aligners, SparkGA, and Halvade. The proposed framework achieved 22.46% more efficient F-score and 99.80% consistent accuracy on average. More, comparatively 0.21% mean higher accuracy is achieved. Moreover, SNP mining has also been performed to identify specific regions in genome sequences. All the frameworks are implemented with the default configuration of memory management. The observations show that all workflows have approximately same memory requirement. In the future, it is intended to graphically show the mined SNPs for user-friendly interaction, analyze and optimize the memory requirements as well.

## 1. Introduction

The knowledge base of biological data can be collected from natural life, scientific experiments, and research archives. Classical organism databases are purposeful where species-specific data are available, as it has great significance in new discoveries. The biological databases have a significant role in bioinformatics as it helps to approach a wide range of biological data along with increased varieties of organisms. Many biological research studies have been piloted and formed significant resources for genomic data. It is often declared that these data resources have not been fully explored yet [1]. These data sources also posture statistical problems; e.g., the family-wise error rate (FWER) [2] shows the occurrence probability of at least one false discovery in multiple tests as it is well known that multiple tests may cause serious false positive problems. The FWER increases with the increase of marker candidates [2,3]. It is investigated that there is a thoughtful issue of computation slant in genomic data, i.e., the size of the input file is the same while processing time of variant calling is still significantly different [4]. Single nucleotide polymorphism (SNP) is a variant of a single nucleotide which exists at a particular locus in the genome, where respective variant exists up-to noticeable degree in a population of a residence [5,6,7,8]. SNP is a genetic variation triggered by the alteration of a single nucleotide base i.e., A, C, G, and T in DNA sequences [9]. It is helpful in biological research to identify the phenotype and genotype properties of individuals. The phenotype is related to physical appearance and genotype is the genetic patterns of an individual. SNPs make an individual different from others as well as helpful to identify the genomic variation in a population. Genomic variation is the key factor to recognize an individual’s relationship to a population. SNPs play an important role to detect genome-based diseases, drug design, the reaction of a drug and defenselessness towards an environmental factor like toxin and risk of evolving disease in a population. The ability of SNPs based noninvasive prenatal testing to identify unrecognized twin [10] and heritability of a general psychopathology factor in children [11].

Next generation sequencing (NGS) technology produces a huge amount of biological data that requires powerful computational devices, large memory and specific hardware and software to manipulate a particular problem i.e., SNPs detection, microarray analysis, and phylogenetic tree construction and analysis [12]. Hadoop is a novel platform and uses Map-Reduce functions that run on any compute cluster in order to provide scalability, reusability, and reproducibility [13]. Hadoop Map-Reduce can also be used for computation and processing to detect the SNPs [12]. Hadoop Map-Reduce is helpful to process NGS data to detect the SNPs with optimized processing time. The study of the genome and related diseases with the help of DNA sequencing is valuable. The emerging research tends to explore the DNA through NGS techniques. NGS technologies have developed quickly and are reforming the genomic research scope and drug design improvements [14]. Identifying genetic variants existing in the genome remains vibrant to explore reasons for phenotype variations and proneness towards cancers and polymorphic infectious viruses. SNPs are commonly used types to explore the genetic variation in the genome. NGS helps to efficiently discover more SNPs with respect to other existing technologies e.g., Sanger sequencing [15].

The Illumina, ABI solid and 454 Life Sciences sequencing technologies have been used in the detection of genomic variation [3,9,16]. The advancement in technologies used for sequencing has produced a huge amount of data in the biological field. But this advancement has given rise issues of the requirement of large memory and computation overhead [17]. The accessibility of technology with high power and the use of genomics and pharma-co-genomics studies of huge populations are generating a huge volume of investigational and medical data, over and above specific database extent in excess of the internet [18]. The storage of big data, preprocessing complexity and investigational analysis of datasets became the key problems, which create a bottleneck in the exploration of pipelines. Handling such big datasets obliges data storing excessive capacity along with facilities of processing, analyzing and sharing. DNA sequencing using NGS technology, the detection of SNPs are being performed to find the variation in genome sequences [19].

With the emergence of technology, the cost of processing has been decreased but the size of data has been exponentially increased [18,20,21]. FaSD has based a binomial distribution-based algorithm and uses mutation likelihood to identify SNPs in NGS data [22]. For FaSD evaluation NGS datasets are taken from the blood-derived ordinary sample and the GBM (Glioblastoma multiforme) tumor sample sequenced in the TCGA project (https://gdc.cancer.gov/resources-tcga-users/). These samples were sequenced on the Genome Analyzer II platform (Illumina, San Diego, CA, USA). All sequences were in FASTQ format (csfastq for SOLiD), mined from the NCBI database of genotypic and phenotypic (dbGap) Sequence Read Archive toolkit (SRA) [23]. After alignment, FaSD model is used to call SNPs. The FaSD-score is calculated to quantify the SNPs likelihood and to determine its equivalent genotype. If FaSD-score meets the cutoff-score then it is declared as SNP at that specific locus and is donated as its equivalent genotype. FaSD uses Bowtie for sequence read’s alignment. FaSD variant caller was developed for sequencing data without molecular barcode. Appropriate analytical methods are needed to take full advantage of the molecular barcode information [24]. Additionally, it requires high processing hardware [25]. Several data mining [26] and machine learning [9] methods developed for SNPs detection in NGS data based on Exhaustive Search Methods, Random Forest, Neural Networks, Support Vector Machines, Regression Models, Bayesian Approaches and or Ant Colony Optimization Approaches. These are powerful methodologies, but prone to infrequent patterns in datasets that tend to produce false positives results [9].

High-performance computing technology is being developed to process genomic data sources and perform computational analysis of life sciences [27]. Many researchers discovered filtering approaches and effective computational algorithms to efficiently detect SNPs [9]. An alternate is cloud computing, as a replacement for owing and conserving the dedicated hardware. Cloud computing provides the Map-Reduce as a parallel computing environment. An open-source implementation of the Hadoop Map-Reduce model is developed for big data analytics, for example NGS data [12]. With the emergence of technologies, the cost of sequencing has decreased but the cost of processing and storage increased, while processing of huge amount of data is challenging. NGS takes input data and processes it to produce output, during the processing that data becomes huge in volume which requires more space and computing resources [28]. Several distributed computing frameworks, e.g., Apache Spark have been developed to provide suitable solutions for addressing the scalability issues of variant calling such as SNPs [29]. A large number of genome analysis tools based on distributed and grid computing framework has been proposed in [29,30]. The framework presented in [30] is used for filtering of large genomic data sets called BAMSI, which is multi-cloud service and flexible in the use of compute and storage resources. The frame presented in [31] called SeqWare Query engine is used for storing and searching genome sequence data. The Genome Analysis Toolkit (GATK) is an effective development and determined exploratory tool used for NGS based on the functional programming of Map-Reduce. GATK is used for accuracy, consistency, CPU and memory effectiveness that allows shared and distributed memory parallelization [32]. Halvade uses Hadoop MapReduce based approach for genome analysis, where the variant calling carried out via chromosome divisions. Due to the noticeable variance in the length of chromosomes, the division may cause load imbalance issue [33,34]. Churchill is a closely unified DNA analysis pipeline and can be implemented for variant calling via HaplotypeCalller or FreeBayes [35,36,37]. The imbalance load created by uneven length of chromosomes can be reduced by using parallel variant calls. However, the problem is still considered as computationally intensive. Authors in [38] use Spark for parallel analysis of genomes. The strategy in the proposed work is simple, but it does not consider the adjacent block overlap. Another tool named GATK4.0 [39] equipped with many tools for analysis of genome data is also based on the Spark framework. The tool supports multi-node and multi-core variant calling with parallelization. The tool demand for high computational resources and memory for large datasets. The shuffle operation causes performance bottlenecks. To address the issue of SNPs detection, the genome sequence analysis pipeline also implemented in parallel through a scalable distributed framework e.g., SparkGA [38]. SparkGA has been widely used with the popularity of big data technology. This implementation is highly capable of parallelizing computation at data-level and highly scalable along with load balancing techniques. GenomeVIP [40] is an open-source platform for the mining of genomic variant discovery, interpretation and annotation running on the cloud and or local high-performance computing infrastructure. Although a number of tools are developed independently, they contain innumerable configuration options and lack of integration which makes it cumbersome for a bioinformatician to use properly. SNPs detection in NGS is critical as its analysis used in many applications like genome-based drug design, disease detection, and microarray analysis. Therefore, more investigations are required to develop a fast, scalable and more accurate SNPs detection framework. In this research study, we proposed a fast and scalable workflow for SNPs detection based on Hadoop Map-Reduce with the integration of Bowtie aligner and parallelized Heap, which enhanced the SNPs detection rate and optimized the execution time. Moreover, mining of SNPs is also introduced in the proposed workflow. The results obtained are compared with state-of-the-art algorithms i.e., GATK [32], FaSD [22], Halvade [33], SparkGA [38], and Heap [8] algorithms.

## 2. Materials and Methods

**Figure 1:**
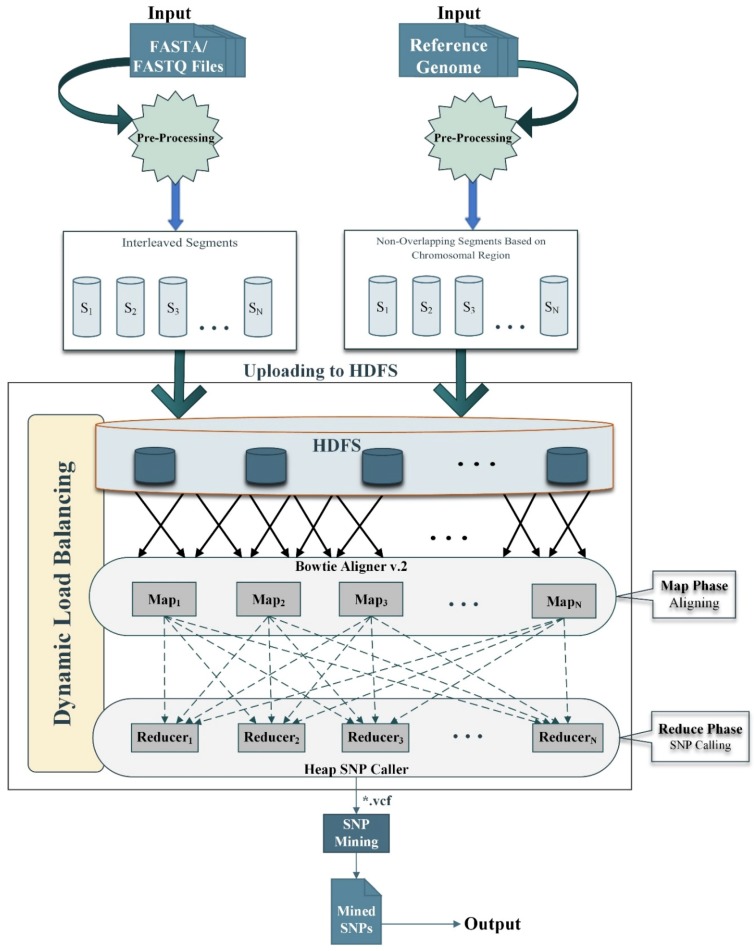
The graphical representation of the proposed workflow. Both target and reference sequences are given as input to the model. Both inputs files are preprocessed as described in Section 2.1. Then the generated segments, i.e., interleaved and non-overlapping segments are uploaded to Hadoop Distributed File System (HDFS) for onward processing. In the map phase, the input data is aligned to the reference genome using Bowtie v.2 aligner as described in Section 2.2. The output of the map phase is collected in a reduce phase for SNPs detection, then Heap is used for detecting the single nucleotide polymorphisms (SNPs) as described in Section 2.3. Finally, the detected SNPs are mined, and the output is generated into a single variant calling format (VCF) file.

**Figure 2:**
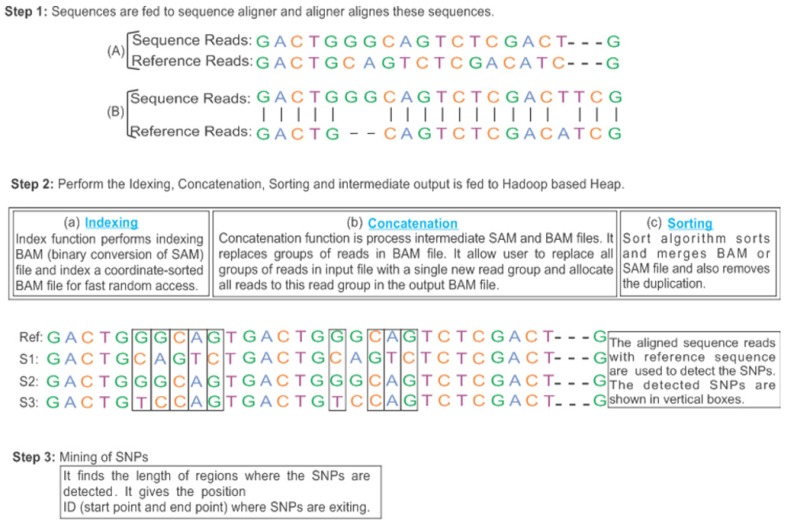
The stepwise procedure of proposed workflow i.e., (**Step 1**): sequences are aligned using the Bowtie aligner version 2. (**Step 2**): Indexing, Concatenation, and Sorting operations are performed then SNP caller is used to detecting SNPs in target sequences in comparison of a reference sequence. (**Step 3**): The mining of detected SNPs is performed to find out the regions where SNPs exist.

**Figure 3:**
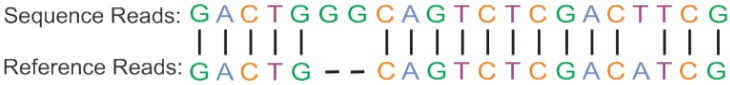
Alignment of target sequence read with reference genome read. The nucleotide of the target sequence read, and a reference sequence is aligned using one-to-one corresponding. The bar sign shows the matched alignment, dashes show the spaces and alignment without bar sign shows the mismatched positions.

This research aims to improve SNPs detection in order to enhance the accuracy rate and optimize execution time. Our proposed framework relies on the Hadoop Map-Reduce programming model [41] which enables parallel and in-memory distributed computation. Hadoop is a free and open-source software platform that is used to process huge amounts of data and run applications in parallel on a cluster environment. It works on divide and conquer based techniques and concludes the results. It consists of a map and reduce functions for processing and Hadoop Distributed File System (HDFS) for storage [13]. Map-Reduce works by breaking the processing into two phases i.e., map phase and reduce phase. The fundamental concept of Map-Reduce is based on *<key, value>* pairs. The map phase takes input in *<key, value>* pairs. It produces the output in the form of a *<key, value>* pairs. The output key-value can be different as compared to the input key-value. The output of various map tasks is group together. The keys and associated set of values are sent to the Reduce phase. The Reduce phase operates on keys and an associated list of values. The output of Reduce is being concatenated and written on HDFS. The proposed framework for SNPs detection using the Map-Reduce paradigm is presented in Figure 1. The stepwise processes are shown in Figure 2; Figure 3 respectively. Moreover, the proposed framework also utilizes a dynamic load balancing algorithm based on [38] with some preprocessing of data format for compatibility to efficiently use the available resources. The proposed model consists of preprocessing, sequence alignment, and SNPs calling and mining integrated with dynamic load balancing as discussed next.

### 2.1. Preprocessing

The FASTA [42] and FASTQ [43] programs are widely used for biological sequences because they are fast, sensitive, and readily available. FASTA and FASTQ have emerged as a common file format for sharing sequencing reads data and are associated with per base quality score. Initially, the segmentation utility [44] which runs on master node locally takes input dataset in FASTA and or FASTQ format to make them accessible for all active computing instances, e.g., map tasks. The segmentation utility creates compressed segments of the default size of the HDFS block, e.g., 64 MB for parallel execution using map tasks. For example, it reads ‘N’ number of blocks in one iteration from a file, where ‘N’ represents the number of map tasks available for execution. Upon reading the specified blocks, each block is assigned to separate map task. All map tasks are executed in parallel to compress the assigned blocks, which are then uploaded to HDFS. The utility used here reads a block of data at once from the input file and looks for the read’s boundary at the end of each block in order to check the ending of last read. The data is taken till the last read and stores the leftover portion in a buffer, which is then appended with next block of incoming data. Meanwhile, the data for a segment is interleaved in map tasks, e.g., a particular map task interleaving data and writing it to segment. Block-by-block reading of dataset is one of the reasons that the proposed model performs significantly better than other programs e.g., Halvade [33,34], which reads the data line-by-line. A status file is also uploaded in order to keep track record of input segments. The status file is used to inform the alignment program that particular segment has been uploaded. The status file contains IDs starting from 0; therefore, segments from 0 to ‘N-1’ will be uploaded first in case if there exist ‘N’ number of map tasks available for execution and the segments from ‘N’ to ‘N × 2-1’ are uploaded next, and so on. When all the segments uploads then a signal in the form of a sentinel file sent to show that all input datasets have been uploaded.

More, some preprocessing steps are also applied to the reference genome prior to the actual execution of Map-Reduce functions, e.g., the reference genome is divided into a preset number of non-overlapping segments. This segmentation is performed on chromosomal regions of approximately equal-sized; where, the chromosomal regions corresponds to the reduce tasks available for execution. The number of reduce tasks can be configured in advance based on the size of the reference genome. Moreover, it is also ensured that all the required data i.e., configuration files and binaries are accessible to each compute node. When all the required data are fetched to each compute node then these preprocessing phases can be ignored. Performing preprocessing on datasets to make them available on each corresponding compute node before actual execution minimizes the overhead of file I/O.

### 2.2. Map Function and Sequence Alignment

The input sequence reads are divided into segments as the default size of HDFS i.e., 64 MB. The Bowtie aligner v.2 [45] is used for aligning reads. Bowtie is a very fast and memory-efficient sequence aligning tool with the existence of reference genome sequences. Bowtie performs chromosome-wise data partitioning and shuffling and aligns the sequence reads with reference reads. It performs the exact matching, which is the foremost feature of Bowtie and helpful to detect more SNPs. In the map phase, each segment is considered as a separate split, hence processed as a single aligner instance. These are parallel executed on each compute node while utilizing all available mappers. Generally, the number of map tasks is much greater than the number of mappers, means that several map tasks will be processed by each mapper. In order to reduce the cost of network communication overhead and to minimize the repeated access of files stored remotely, our proposed model preferably makes use of map tasks that have locally input segments as part of HDFS. The indexing, concatenation, and sorting functions are based on Hadoop BAM [46] as shown stepwise in Figure 2. Hadoop BAM utilizes the Java libraries to manipulate the files in communal bioinformatics formats through the Hadoop Map-Reduce framework along with Picard SAM JDK as well as command-line tools, e.g., SAM-tools. Hadoop BAM is a novel library for the scalable manipulation and aligning next-generation sequencing data in the Hadoop distributed computing framework. The genome reads are parsed through Hadoop-BAM and aligned to the reference genome as shown in Figure 3, that are already available on each compute node. It acts as an integration layer between analysis applications and BAM files that are processed using Hadoop. Hadoop BAM solves the issues related to BAM data access by presenting a convenient API for implementing map functions that can directly operate on BAM records. It builds on top of the Picard SAM JDK, so tools that rely on the Picard API are easily convertible to support large-scale distributed processing. Upon successful completion of all alignments, the reads are transformed to *<key, value>* pairs, where each key is generated from SAM records i.e., *<id_chromosomal_region, position_of_mapping>*; the key shows the exact location (mapping position) of mapping in the reference genome. Index function performs indexing BAM (binary conversion of sequence alignment map [SAM]) file and index a coordinate-sorted BAM file for fast random access. The concatenation function processes intermediate SAM and BAM files. It replaces groups of reads in the BAM file. It allows us to replace all groups of reads in the input file with a single new read group and allocate all reads to this reading group in the output BAM file. Sort algorithm sorts and merges BAM or SAM file and removes the duplicate reads. Genome reads that are aligned to the same chromosomal region are grouped together to form a single reduce task.

### 2.3. Reduce Function and Genome Single Nucleotide Polymorphisms (SNPs) Calling

Generally, the number of reduce tasks as much greater than the number of reducers; a number of reduce tasks are executed parallel. A particular task accepts all sorted intermediate *<key, value>* pairs as input for the single chromosomal region, which is stored in SAM or BAM file format. Here, multiple instances are created to perform SNPs calling. Heap is an accurate and highly sensitive SNP detection tool for high throughput sequencing data and offers equally dependable SNPs with distinct locus to genomic prediction (GP) and genome-wide association studies (GWAS) [8]. Heap performs the read filtering in order to obtain a high-quality score based on Phred-scale as shown in Equations (1) and (2). The reads having scored less than 20 and the bases with a score less than 13 are removed from the search scope of valid SNP calling sites. Based on quality filtering the frequency of an allele is computed on all nucleotide sites in order to determine genotype sampling. Heap then performs actual SNPs calling while comparing the genotypes between the reference genome and sample available at each compute node. The reducer function extracts the keys and associated values. It mines the bases A, T, C, and G through the utilization of a fast algorithm for statistical assessment of very large-scale databases [47], that fundamentally one time executes the itemset mining algorithm, while the other algorithms execute several times. Then, counts each base in reading and check either match or mismatch with the corresponding reference sequence. It also maintains the definite record based on the base quality which is very helpful to realign and recall of SNPs if detection accuracy remains inconsistent. The reduce function to release the *<key, value>* pairs. Variant calling format (VCF) file is generated at the end of each reduce task. VCF file consists of the SNPs detected in the corresponding chromosomal region. Finally, all the VCF files are merged into a single VCF file to present all the SNPs detected among the samples. The mining of SNPs generates the output to show the region-wise saturation. SNP caller calls the SNPs and generates the output which provides the number of SNPs. This study has improved the SNP caller results and SNP mining which shows the specific positions in the genome where the SNPs exist. It is more helpful for the target-based investigation of SNPs in a specific range of a genome.

(1) $$P=10^{\frac{-Q}{10}}$$

where *P* represents error probability

(2) $$Q=-10{\;\log}_{10}P$$

where *Q* represents quality score (Phred Score).

### 2.4. Dynamic Load Balancing

In order to get the best performance from available resources, a dynamic load balancing algorithm as shown in Algorithm 1 is applied to balance the load, which remains active in process execution. A region with too many reads can be further divided via dynamic load balancing, so the execution time for several procedures in the workflow depends on the number of reads being processed. It is used as a local resource manager and is responsible for managing computing resources. Particularly, the dynamic load balancing algorithm consists of load estimation and resource management components. The load estimation component is used to calculate a load of a task instance while considering the size of data and training parameters, which are used to represent the computational complexity. The resource management component is used to assign the estimated amount of resources physically. It is worthful to note here that the dynamic load balancing algorithm does not change the resource scheduling algorithm of the Hadoop framework. Rather, it takes over the resources that have been pre-assigned for each lunched task. Then, the dynamic load balancing algorithm is used to re-assign the resources for sub-constitute tools in each task via reconfiguring their runtime parameters.
**Algorithm 1.** Dynamic load balancing algorithm
**Procedure_generate_BAM_files****1.****Elements of the distributed dataset {region_id, (region_id, (segment_id, number_of_reads)}**
Input ← seq_read_info (dynamic_load_balancing_info).cache()**2.****Obtain the number of sequence reads for each segment**
Seq_reads_per_segment ← input.map[(region_id,{segment_id, number_of_reads)} => number_of_reads**3****Obtain the total number of sequence reads**
Total_number_of_reads ← number_of_reads_per_segment.reduce_by_key()**4.****Obtain chromosomal region-based input data and group them**
Chromosomal_region ← input.group_by_key() Chromosomal_region.cache()**5.****Compute the average number of reads based on load balancing region**
Avg_seq_reads ← total_reads/chromosomal_region.count()**6.****Generate BAM to further divide a region (if required) and build BAM files**
Chromosomal_region.foreach{(region_id, list_info) → generate_BAM(region_id,list_info, avg_seq_reads_region)}
**end procedure**

### 2.5. Experimental Setup

Experimental datasets are obtained from NCBI [23] and DDBJ DRA [48,49] web portals, which provide free access to biomedical and genomic data along with verified statistics. Two benchmark datasets are selected for experiments based on compatibility of parameters, e.g., Sorghum and the human genome. Three datasets of Sorghum e.g., GULUM_ABIA (DRR045054), RTx430 (DRR045061), SOR 1 (DRR045065) consist of 1,573,011, 2,251,325, and 2,942,974 number of reads respectively. The number of base pairs in each dataset is 158,874,111, 227,383,825, and 297,240,374 respectively. Each dataset consists of 1,000,000 genome length and 101 read length. The reference genome Sbicolor_v2.1_255 is used for Sorghum datasets. The human genome dataset NA12878 consists of 1.6 billion 101 bp paired-end reads stored in two FASTQ files of 97 GB in size compressed with gzip compression tool (https://www.gzip.org/). The human genome hg19 resource bundle available from [50] is used for reference. For results visualization and ease of understanding the results obtained for both datasets are separately plotted, while same parameters and experimental setup is used for comparison and analysis.

Various experimental setups are used for the evaluation of the proposed framework in comparison with other state-of-the-art models e.g., GATK 4.0, FaSD, Halvade, and SparkGA. Single node pseudo cluster and real clusters consist of 8, 16, and 32 working nodes are used for scaling and analysis. Single node pseudo cluster consists of Intel^®^ Core™ i7-7700K with four cores @ 4.20 CPU having eight threads along with 64-GB of memory installed, running on 64-bit instruction set kernel Linux (Ubuntu 16.04.6 LTS) operating system (OS). The real clusters comprise of 8, 16, and 32 compute nodes, the machine used in single node pseudo cluster are configured as server and rest of each node consist of Intel(R) Core i5-7600K with four cores @ 3.8 GHz CPU, 16-GB of memory installed, running on 64-bit instruction set kernel Linux (Ubuntu 16.04.4 LTS) OS. All the nodes are connected through the 10Gbit/s Ethernet network.

### 2.6. Measurement Metrics

Sensitivity, Specificity, and Accuracies are the terms that are mostly associated with a classification test and they statistically measure the performance of the test. In classification, we divide a given data set into two categories based on whether they have common properties or not by identifying their significance in a classification test. In general, sensitivity indicates, how well the test predicts one category and specificity measures how well the test predicts the other category. Whereas Accuracy is expected to measure how well the test predicts categories. If an SNP detected, further it has two possibilities as either it is true or not which is termed as a true positive (TP) and false positive (FP) respectively. Similarly, on the other hand, if an SNP is not detected, then it has also two categories i.e., true negative (TN) or false negative (FN). In [8], true detection of SNPs is based on sensitivity, positive predictive value (PPV), F-score, and accuracy. With the use of efficient SNPs detection algorithmic solution, the rate of TP and TN helps to increase the F-score and accuracy rate. The detected SNPs through GATK, FaSD, and Heap SNPs caller along with the integration of BWA and Bowtie aligner, SparkGA and Halvade are compared with the results of proposed framework i.e., Hadoop based Heap SNP caller integrated with Bowtie aligner. The F-score and accuracy of SNP callers are also recorded, where TP, FP, FN, TN, and PPV are considered as standard measurement parameters. The computational processes of chosen parameters are presented in Equations (3)–(6).

(3) $$\mathrm{Sensitivity}=\frac{\mathrm{TP}}{\left(\mathrm{TP}+\mathrm{FN}\right)}$$

(4) $$\mathrm{PPV}=\frac{\mathrm{TP}}{\left(\mathrm{TP}+\mathrm{FP}\right)}$$

(5) $$F-\mathrm{score}=2\times \frac{\mathrm{PPV}*\mathrm{Sensitivity}}{\left(\mathrm{PPV}+\mathrm{Sensitivity}\right)}$$

(6) $$\mathrm{Accuracy}=\frac{\mathrm{TP}+\mathrm{TN}}{\left(\mathrm{TP}+\mathrm{FP}+\mathrm{TN}+\mathrm{FN}\right)}$$

**Figure 4:**
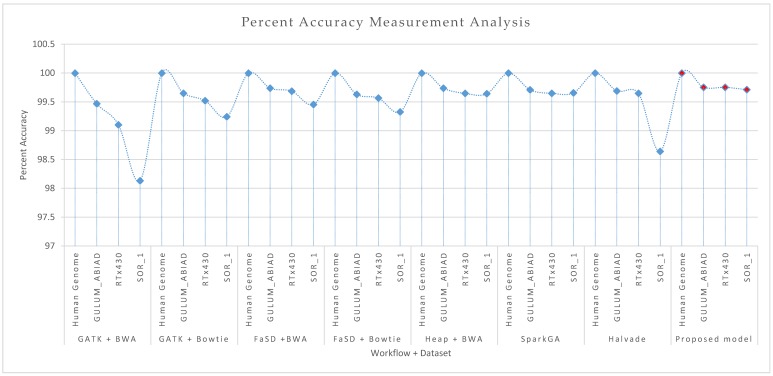
Comparative results analysis of proposed and existing workflows in terms of percent accuracy measurement on the Human genome, GULUM_ABIAD, RTx430, and SOR_1 datasets. The figure shows the results for GATK + BWA, GATK + Bowtie, FaSD + BWA, FaSD + Bowtie, Heap + BWA, SparkGA, Halvade and proposed workflows respectively. The diamond shape marker over the dotted line shows the discrete points for the accuracy of the respective workflow and dataset. The light blue marker shows the existing workflows, while the solid red filled marker shows the points for the proposed workflow. Results analysis of graphs shows that the proposed workflow comparatively outperforms than existing workflows.

**Figure 5:**
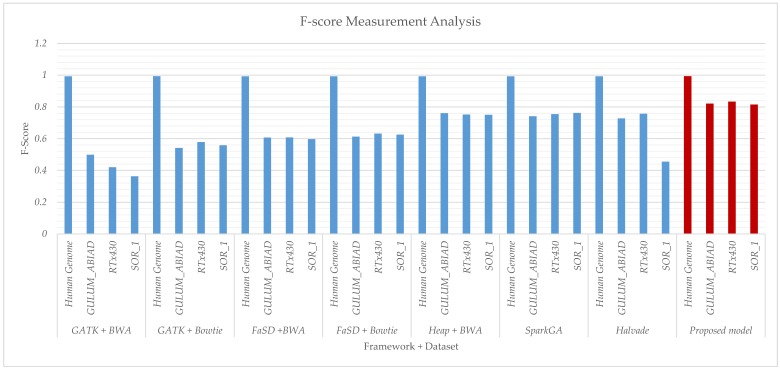
Comparative results analysis of proposed and existing workflows with respect to F-Score executed on the human genome and Sorghum datasets GULUM_ABIAD, RTx430, and SOR_1. The graph shows the F-score results for GATK + BWA, GATK + Bowtie, FaSD + BWA, FaSD + Bowtie, Heap +BWA, SparkGA, Halvade and proposed workflows respectively. The solid blue bars show the F-score for existing workflows while the solid filled dark-red vertical bars show the F-score result for the proposed workflow. From graphs, it is clear that the proposed workflow has achieved better results as compared to others.

Table 1 shows the empirical results of F-score and accuracy for all algorithms and respective datasets used. Figure 4 and Figure 5 show the comparative results of accuracy and F-score for all frameworks respectively. The frameworks GATK and FaSD are integrated with BWA and Bowtie aligners. Results show that the Bowtie aligner produces better results than BWA in terms of F-score while the accuracy of BWA is better than the Bowtie aligner. Heap SNP caller is then integrated with BWA aligner and results are recorded for comparison. The comparative analysis of Heap integrated with BWA shows better results than GATK and FaSD integrated with BWA and Bowtie aligners. The SparkGA model is also executed, where its results are slightly better than previous frameworks. The Halvade framework results are also compared with other frameworks, however, its results are not significant on selected parameters. The results analysis of the proposed framework shows that it outperforms than existing algorithms in terms of parameters used in the comparison.

### 2.7. Single Nucleotide Polymorphism (SNP) Mining

Most of the SNPs caller algorithm detects the SNPs and generates the output in VCF file format. The output shows the details of SNPs detected and the number of SNPs. SNPs mining facilitates to identify the region-wise position of SNPs throughout the genome length in terms of position ID. The ID contains the starting position and ending position of a genomic region where SNPs exist. The region length tells the length of the region of these SNPs.

## 3. Results and Discussion

To evaluate the correctness and validity of the proposed framework sample datasets were extracted from all benchmarked datasets with consistent length i.e., 2000 genome length with 101 read length and executed on a single node. Each workflow experiment was executed 100 times and average time in seconds is computed for sample datasets, where the results of real clusters are recorded in minutes for clear visualization and ease of understanding. Results analysis of sample datasets shows that the proposed framework produced good results than others. For scalability analysis all the workflows are evaluated on real compute clusters of different configurations i.e., 8 compute nodes @ 116 GHz processing power with 32 cores equipped with 112 GB of memory, 16 compute nodes @ 237.60 GHz processing power with 64 cores equipped with 304 GB of memory and 32 compute nodes @ 471.2 GHz processing power with 128 cores equipped with 560 GB of memory. All the nodes are connected through 10 Gbit/s Ethernet network. GATK correctly calls SNPs if enough numerals of reads coverage are delivered i.e., 20× or more for enough sensitivity in genome re-sequencing, which is difficult under low read coverage, 7× or lower. FaSD uses the Bowtie for sequence read’s alignment by default. Additionally, it requires high processing hardware infrastructure. Heap improves the sensitivity and accuracy of SNPs calling with lower coverage NGS data. Heap reduces the FP rate and accomplishes the highest F-scores with low coverage (7×). F-score is the harmonic means of sensitivity and PPV.

The default configurations for memory utilization and management are considered for all the existing workflows. For a fair comparison, the default configuration for memory management of Hadoop Map-Reduce is also considered for the proposed model as described next. On every node, Map-Reduce updates mapred-site.xml file with the number of map and reduce slots based on the number of computing instances available on the node. Traditionally, data are stored in block units. The memory path is updated upon the writing of each data block and finally reaches to the end of the array for redirection to the head. To make sure that data are written into the memory, the policy is re-written for selecting the storage path in the HDFS. Data files are assigned paths with different priorities, sort them based on priority, and then store the file paths into the array of the data node and check the paths in the array from the start when data is written. The observations and analysis of memory utilization show that all the workflows including the proposed model consume approximately the same memory.

GATK uses the BWA aligner as the default aligner, however, in [51] the GATK’s results are reviewed and generated using Bowtie aligner which improves the results with respect to SNPs calling. Similarly, FaSD uses the Bowtie aligner as default, while in [52] the performance of FaSD with respect to SNPs calling using the BWA and Bowtie aligner are presented. The integration of Bowtie with FaSD produces more improved results than BWA as GATK integrated with Bowtie aligner. Heap uses the BWA for sequence alignment as the default aligner. We have integrated the Bowtie aligner with Heap and executed on Hadoop clusters and get improved results.

Results given in Figure 4 and Figure 5 show the accuracy and F-score measurement analysis of the proposed framework in comparison with GATK + BWA, GATK + Bowtie, FaSD + BWA, FaSD + Bowtie, Heap + BWA, SparkGA, and Halvade pipelines respectively. Results analysis show that the proposed model is 52.3%, 29.6%, 23.4%, 20.9%, 6.3%, 6.5%, and 18% more efficient in F-score than GATK + BWA, GATK + Bowtie, FaSD + BWA, FaSD + Bowtie, Heap + BWA, SparkGA and Halvade pipelines respectively. It also shows that the proposed framework is 0.63%, 0.20%, 0.08%, 0.17%, 0.04%, 0.05%, and 0.31% more accurate than GATK + BWA, GATK + Bowtie, FaSD + BWA, FaSD + Bowtie, Heap + BWA, SparkGA and Halvade pipelines respectively. Results from Table 1 and Figure 4 show that the proposed model achieved 99.998% accuracy on the human genome, 99.75% on GULUM_ABIAD, 99.75% on RTx430, and 99.71% on the SOR_1 dataset, its analysis show that the proposed framework is consistent for accuracy gain as compared to others. The overall analysis of Figure 4; Figure 5 shows that the proposed framework is 22.46% more efficient and 0.21% more accurate on average empirical observations comparatively.

**Figure 6:**
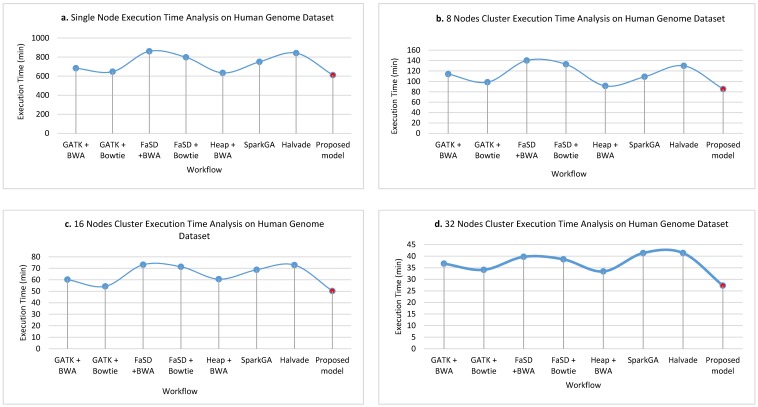
The execution time of single node pseudo cluster, 8 nodes, 16 nodes and 32 nodes real clusters are shown in part (**a**–**d**) respectively for the human genome dataset. The light blue filled circles over the line show the points for existing workflows and the solid red filled circle shows the values for the proposed workflow. Its analysis shows that the proposed model takes less time in execution while detecting SNPs in the human genome dataset.

Figure 6a–d show the execution time taken on the human genome dataset while running on a single node, 8 nodes, 16 nodes, and 32 nodes cluster respectively. The graphs show the comparative results of the proposed framework and GATK + BWA, GATK + Bowtie, FaSD + BWA, FaSD + Bowtie, Heap + BWA, SparkGA, and Halvade pipelines executed for human genome dataset. The results analysis shows that the proposed framework outperforms on the human genome dataset than others. It shows that the proposed framework gained the benefit of scalability power of Hadoop Map-Reduce. It achieved high efficiency in terms of execution time on the human genome as it has a large number of bases. While other workflows remain inconsistent in execution time and have a varying span of execution time on the human genome dataset. The proposed framework utilizes the power of Hadoop Map-Reduce integrated with Heap along with Bowtie aligner.

**Figure 7:**
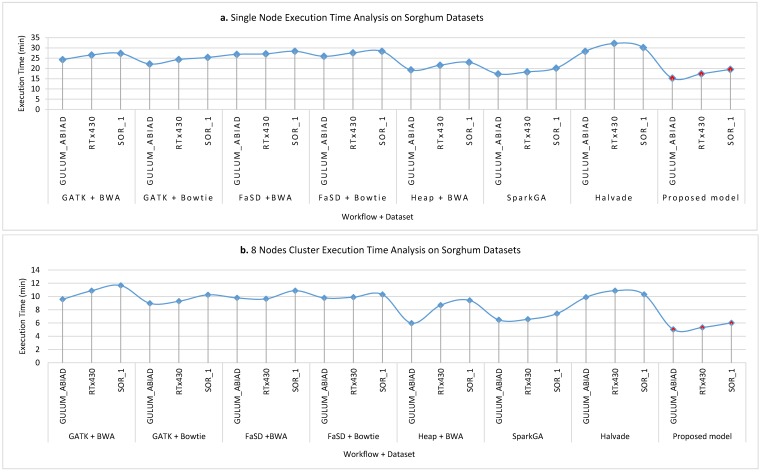

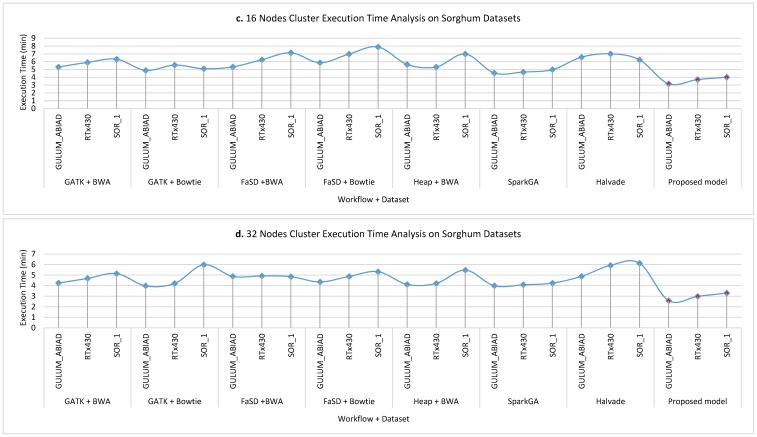
The execution time analysis of single node pseudo cluster, 8 nodes, 16 nodes, and 32 nodes real clusters are shown in part (**a**–**d**) respectively for Sorghum datasets. The light blue filled circle markers over the line show the values for existing workflows and the solid red filled circle shows the values for the proposed workflow. Here, it is clear that the proposed workflow takes less time in execution for detecting SNPs as compared to others. It is worth noting that on a larger dataset the efficiency of the proposed framework is much better than a smaller dataset.

Figure 7a–d show the execution time taken on Sorghum datasets while running on a single node, 8 nodes, 16 nodes and 32 nodes cluster respectively for all workflows. The analysis of results shows that the proposed model takes less time as compare to others. However, it is observed that the proposed framework gain less efficiency as compared to efficiency gain over the human genome dataset. The reason behind this the size of the dataset and the logic of Hadoop Map-Reduce execution. The communication overhead cost of other pipelines is much more than the proposed model because the proposed model utilizes the dynamic load balancing algorithm for maintaining the load balanced throughout the execution of the workflow.

**Figure 8:**
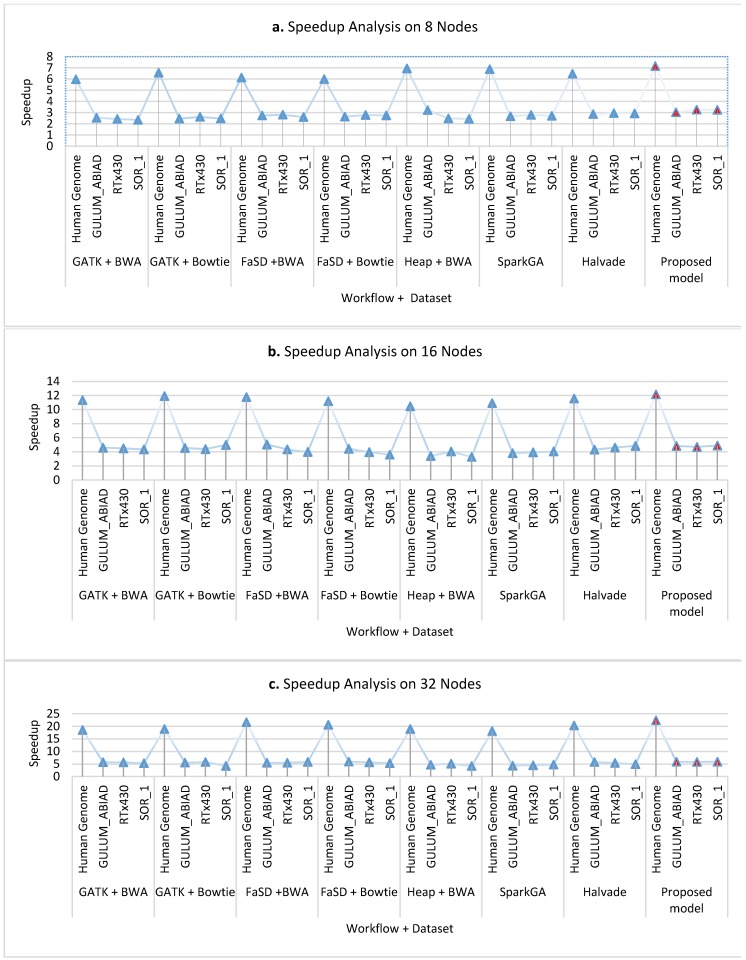
The graphs (**a**–**c**) show the speed up analysis of the proposed workflow in comparison with existing workflows running on various nodes: 8, 16, and 32 nodes respectively. On all number of nodes, the proposed workflow achieved higher speedup as compared to other workflows on both Sorghum and Human genome datasets. Results analysis elucidates that the proposed workflow is robust in gaining speed up.

**Figure 9:**
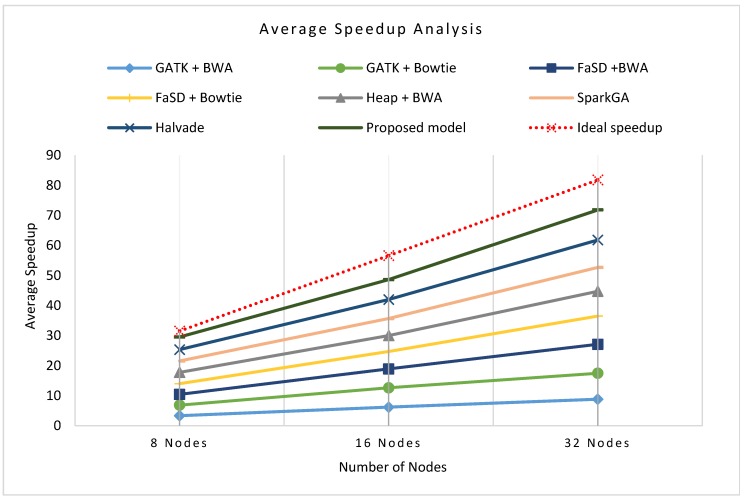
Average speed up analysis of the proposed workflow in comparison with existing workflows on a number of various nodes. The graphical representation shows that the proposed workflow achieved higher speedup performance on average of all clusters and datasets as compared to other frameworks.

Figure 8a–c presents the cluster-wise speedup gained by the proposed model over other workflows while running on 8 nodes, 16 nodes, and 32 nodes clusters respectively for all datasets. The scalability analysis of all workflows show that the proposed framework is highly scalable as it has achieved good speedup on 8, 16, and 32 compute nodes. Figure 9 shows the average speedup measurement analysis of 8, 16, and 32 nodes real compute clusters for all datasets its analysis presents that the proposed framework outperforms than others on all datasets.

## 4. Conclusions

SNP is a variation of a single nucleotide that exists at a particular locus in the genome, where respective variant exists to a noticeable degree in the population of a residence. Detecting SNPs in high dimensional genomic data is difficult, due to the growing number of genetic variations in genome sequences. It is helpful in biological research to assess an individual’s reaction to certain drugs, defenselessness towards environmental factors like toxins, and risk of disease. The Hadoop is a novel platform using the Map-Reduce programming framework which runs on any cluster only with the prerequisite of Java. It provides the scalability, reusability, and reproducibility features. The Hadoop Map-Reduce can also be used for fast computation and processing to detect the SNPs in genome sequences. Hadoop Map-Reduce proves the capability to process NGS data to detect the SNP in less time with higher accuracy. In this research study, we proposed Hadoop based framework integrated with Heap for SNPs detection which enhances the SNPs detection rate and optimizes the execution time. The proposed framework is executed on a various number of nodes with different configurations. To validate the framework, different benchmark datasets have been used and the results are recorded for comparison with other state-of-the-art pipelines. This research contributed as a novel framework for SNP detection which has improved the SNPs detection rate, optimized the execution time and mined SNPs as well.

In the future, it is intended to identify SNPs associated with complex diseases such as cancer, diabetes, and heart disease on a large scale, e.g., cloud computing environment integrated with optimization technique of artificial intelligence and mine them. It is also intended to optimize memory requirement in the future.

## Figures and Tables

**Table 1:** Comparative measurement analysis of proposed workflow with other frameworks in terms of mean F-Score and mean accuracy. Bold is used to highlight the proposed method and its results.

Algorithm	Datasets	TP	FP	FN	TN	PPV	Sensitivity	F-Score	Accuracy %	Mean F-Score	Mean Accuracy
Genome Analysis Toolkit (GATK) + BWA	Human Genome	4,217,638	42,983	20,871	2,995,718,508	0.98991156	0.99507586	0.992487	99.9978715	0.56823916	99.1739429
	GULUM_ABIAD	2649	421	4912	992,018	0.86286645	0.35035048	0.49835387	99.4667		
	RTx430	3248	763	8229	987,760	0.80977312	0.28300078	0.41942149	99.1008		
	SOR_1	5320	1281	17,415	975,984	0.80593849	0.23400044	0.3626943	98.1304		
GATK + Bowtie	Human Genome	4,218,954	41,768	17,524	2,995,721,754	0.99019697	0.99586355	0.99302217	99.9980236	0.66755026	99.6017809
	GULUM_ABIAD	2070	1242	2269	994,419	0.625	0.47706845	0.54110574	99.6489		
	RTx430	3284	1019	3778	991,919	0.76318847	0.46502407	0.57791465	99.5203		
	SOR_1	4801	2188	5413	987,598	0.68693661	0.47004112	0.55815846	99.2399		
FaSD +BWA	Human Genome	4,217,748	40,158	25,474	2,995,716,620	0.9905686	0.99399654	0.99227961	99.9978123	0.70102669	99.7179531
	GULUM_ABIAD	2036	1778	856	995,330	0.53382276	0.70401107	0.60721742	99.7366		
	RTx430	2438	2334	817	994,411	0.5108969	0.74900154	0.60744986	99.6849		
	SOR_1	4058	3176	2299	990,467	0.56096212	0.63835142	0.59715989	99.4525		
FaSD + Bowtie	Human Genome	4,218,962	41,718	19,874	2,995,719,446	0.99020861	0.99531145	0.99275347	99.9979469	0.71574353	99.6295367
	GULUM_ABIAD	2933	1170	2539	993,358	0.7148428	0.53600146	0.61263708	99.6291		
	RTx430	3739	1162	3185	991,914	0.76290553	0.54000578	0.63238901	99.5653		
	SOR_1	5623	1855	4887	987,635	0.75193902	0.53501427	0.62519457	99.3258		
Heap + BWA	Human Genome	4,219,867	41,875	19,724	2,995,718,534	0.99017421	0.99534766	0.9927542	99.9979467	0.81385601	99.7553367
	GULUM_ABIAD	4183	1611	1026	993,180	0.72195375	0.80303321	0.76033809	99.7363		
	RTx430	5352	2251	1279	991,118	0.70393266	0.80711808	0.75200225	99.647		
	SOR_1	5408	2231	1368	990,993	0.70794607	0.79811098	0.75032952	99.6401		
SparkGA	Human Genome	4,217,945	42,893	17,890	2,995,721,272	0.9899332	0.99577651	0.99284626	99.9979739	0.81260408	99.7523685
	GULUM_ABIAD	4189	1745	1180	992,886	0.70593192	0.78021978	0.74121915	99.7075		
	RTx430	5395	2358	1157	991,090	0.69585967	0.8234127	0.75428172	99.6485		
	SOR_1	5517	2198	1247	991,038	0.71510045	0.81564163	0.7620692	99.6555		
Halvade	Human Genome	4,215,987	42,598	18,574	2,995,722,841	0.98999715	0.99561371	0.99279749	99.9979609	0.73330211	99.4933902
	GULUM_ABIAD	4171	1869	1247	992,713	0.69056291	0.76984127	0.72805027	99.6884		
	RTx430	5487	2498	1024	990,991	0.68716343	0.84272769	0.75703642	99.6478		
	SOR_1	5687	2359	11,247	980,707	0.70681084	0.33583323	0.45532426	98.6394		
**Proposed model**	**Human Genome**	**4,325,715**	**41,350**	**17,865**	**2,995,615,070**	**0.9905314**	**0.99588703**	**0.993202**	**99.9980262**	**0.86552278**	**99.8031315**
	**GULUM_ABIAD**	**5680**	**1458**	**1024**	**991,838**	**0.7957411**	**0.84725537**	**0.82069065**	**99.7518**		
	**RTx430**	**6214**	**1458**	**1025**	**991,303**	**0.80995829**	**0.85840586**	**0.83347864**	**99.7517**		
	**SOR_1**	**6354**	**1532**	**1358**	**990,756**	**0.80573168**	**0.82391079**	**0.81471984**	**99.711**		

True positive (TP), false positive (FP), false negative (FN), true negative (TN), positive predictive value (PPV).
